# Extra-Articular Manifestations and Comorbidities in Psoriatic Disease: A Journey Into the Immunologic Crosstalk

**DOI:** 10.3389/fmed.2021.737079

**Published:** 2021-09-23

**Authors:** Lucia Novelli, Ennio Lubrano, Vincenzo Venerito, Fabio Massimo Perrotta, Francesca Marando, Giacomo Curradi, Florenzo Iannone

**Affiliations:** ^1^Medical Department, AbbVie srl, Rome, Italy; ^2^Department of Medicine and Health Sciences “Vincenzo Tiberio”, University of Molise, Campobasso, Italy; ^3^Rheumatology Unit—Department of Emergency and Organ Transplantations, University of Bari “Aldo Moro”, Bari, Italy

**Keywords:** psoriatic arthritis, psoriatic disease, comorbidities, extra-articular manifestations, systemic inflammation, immunomodulation

## Abstract

Psoriatic arthritis (PsA) is a chronic inflammatory disease primarily affecting peripheral and axial joints, with the possible presence of extra-articular manifestations (EAMs), such as psoriasis, uveitis, and inflammatory bowel disease. Recently, the concept of psoriatic disease (PsD) has been proposed to define a systemic condition encompassing, in addition to joints and EAMs, some comorbidities (e.g., metabolic syndrome, type II diabetes, hypertension) that can affect the disease outcome and the achievement of remission. EAMs and comorbidities in PsA share common immunopathogenic pathways linked to the systemic inflammation of this disease; these involve a broad variety of immune cells and cytokines. Currently, various therapeutics are available targeting different cytokines and molecules implicated in the inflammatory response of this condition; however, despite an improvement in the management of PsA, comprehensive disease control is often not achievable. There is, therefore, a big gap to fill especially in terms of comorbidities and EAMs management. In this review, we summarize the clinical aspects of the main comorbidities and EAMs in PsA, and we focus on the immunopathologic features they share with the articular manifestations. Moreover, we discuss the effect of a diverse immunomodulation and the current unmet needs in PsD.

## Introduction

Psoriatic arthritis (PsA) is a common chronic inflammatory disease characterized by the association of arthritis and psoriasis, first identified by Verna Wright and his colleagues as a distinct and peculiar condition belonging to the group of spondyloarthritis (SpA) (1). Immune dysregulation, with altered cytokines expression and cellular phenotypes is responsible of the typical clinical features of PsA, which involve peripheral joints and the axial skeleton, with the onset of peripheral arthritis and spondylitis (2). In addition to these characteristic musculoskeletal manifestations, patients with PsA can often suffer from extra-articular manifestations (EAMs), which are genetically and immunologically correlated to these features and include psoriasis; inflammatory bowel diseases (IBDs), such as ulcerative colitis (UC) and Crohn disease (CD); and uveitis (3). Moreover, other concomitant or subsequent diseases, globally called comorbidities, can develop in these patients. These conditions are highly prevalent among patients with PsA and can be due to shared genetic/immunologic risk factors, chronic inflammation, the consequences of its treatment, and reduced physical function and activity (Table 1) (4). In recent years, the concept of psoriatic disease (PsD) emerged among the rheumatologic community, defining PsA as a systemic disease encompassing EAMs and comorbidities other than skin and joint involvement (13). This concept was developed as an attempt to explain more fully the complexity of this disease. The presence of EAMs and comorbidities strongly affects disease burden, often correlating with a poorer outcome, worse quality of life, reduced physical function, and poor response to treatments (14). Moreover, EAMs and comorbidities should drive therapeutic choices, and finding the correct balance between disease control, global efficacy, contraindications, and side effects is still a challenge and an unmet medical need, despite the various therapeutics currently available. Herein, we discuss the concept of PsD, reviewing the main PsA comorbidities and EAMs and focusing on some of their shared immunopathologic features and the effect of their modulation in clinical practice.

**Table 1:** Prevalence of comorbidities and EAMs in PsA.

**Comorbidity**	**EAM**	**Prevalence, %**	**References**
CV disease	Psoriasis	19; 7–40	(4, 5)
Metabolic syndrome	IBD	29; 0–29	(3, 4)
Diabetes	Uveitis	6-20; 2–25	(3, 6)
Fibromyalgia	–	17.8–54	(7)
Depression	–	9–22	(8)
Anxiety	–	15–30	(8)
Osteoporosis	–	1.4–68.8	(9–12)

*CV, cardiovascular; EAM, extra-articular manifestation; IBD, inflammatory bowel disease; PsA, psoriatic arthritis*.

## The Concept of Psoriatic Disease

**Figure 1:**
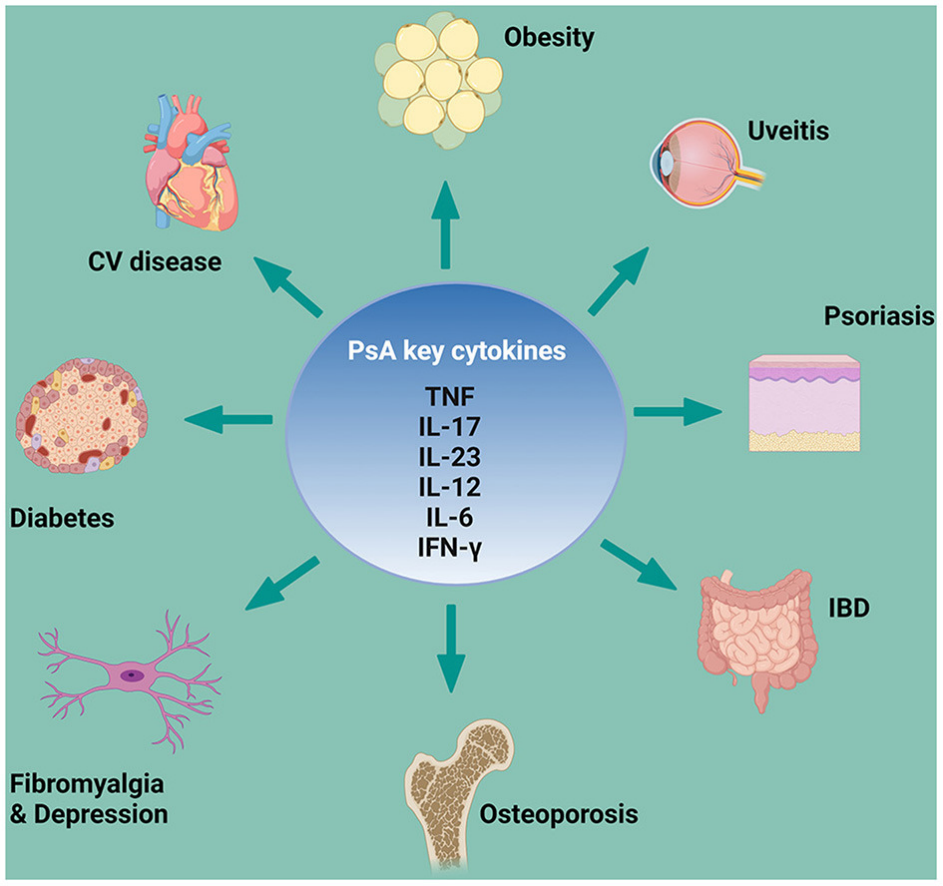
PsA key cytokines involved in comorbidities and EAMs pathogenesis. CV, cardiovascular; EAM, extra-articular manifestation; IBD, inflammatory bowel disease; IFN-γ, interferon; IL, interleukin; PsA, psoriatic arthritis; TNF, tumor necrosis factor. *Created with* *Biorender.com*.

Since the first descriptions of clinical manifestations, PsA appeared to be a multifaceted disease; in 2006, the term PsD was proposed by Scarpa and colleagues to emphasize the clinical and pathogenetic heterogeneity of PsA (13). PsD represents a heterogeneous, chronic, inflammatory disease with a wide spectrum of phenotypical manifestations that can occur only at joint level or in combination with several cutaneous, periarticular EAMs and different comorbidities, (15) which share key cytokines pathways (Figure 1). In this context, patients with PsA may have different clinical phenotypes and presentations, and physicians may need to treat patients in whom several coexisting conditions could be present (16).

## Psoriatic Arthritis Extra-Articular Manifestations

### Psoriasis

Psoriasis (PsO) is the most common non-musculoskeletal organ involvement in PsA. Approximately one-third of patients with PsO develops PsA over time. The incidence rate of PsA in different psoriasis cohorts is between 1.3 and 3.5% per annum (5, 17), with skin involvement preceding arthritis by an average of 7 years (17). PsA mainly develops in patients with an established diagnosis of PsO; indeed, PsO seems to occur after the onset of arthritis only in 15% of PsA cases. Simultaneous onset of PsA and PsO occurs in about 15% of patients, who tend to experience combined flares of both articular and skin involvement (17, 18). This makes PsO the most readily identifiable marker that confers risk of arthritis. Moreover, a recent study found that PsO is more common in PsA patients with axial involvement as well as other EAMs (e.g., uveitis and IBDs) (19). Different clinical subsets of PsO exist, such as plaque psoriasis, nail psoriasis, scalp psoriasis, palmoplantar psoriasis, and inverse or intertriginous psoriasis with distinct morphologic phenotype (5, 20, 21). In particular, nail, scalp and inverse psoriasis has been associated with an increased risk of PsA (5, 22).

**Figure 2:**
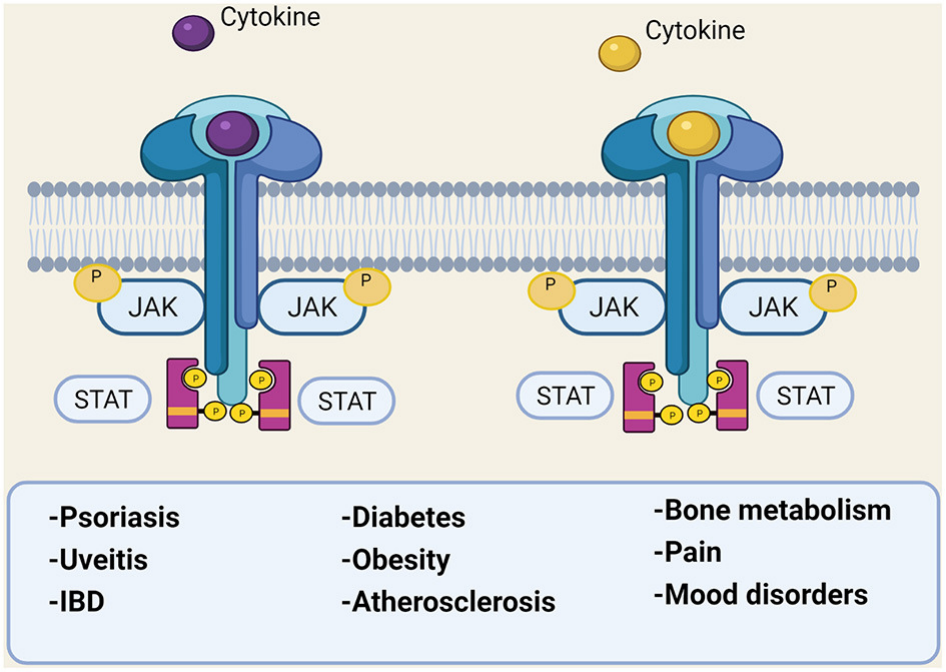
The JAK/STAT pathway involvement in different diseases. IBD, inflammatory bowel disease; JAK, Janus kinase; P, phosphate; STAT, signal transducer and activator of transcription. *Created with* *Biorender.com*.

For many years, PsO has been considered a classical Th1-mediated disease, with a central role of interleukin (IL−2) and interferon (IFNγ); subsequently, other populations of T-helper cells and their cytokines have been identified as major drivers in PsO development (23). First tumor necrosis factor (TNF) and then IL-17 and IL-12/IL-23 pathways have been recognized as key pathogenic circuits in PsO, acting in synergy for the inflammatory cascade. The currently embraced pathogenic model for PsO suggests a trigger event, leading to the release of autoantigens from skin cells (e.g., LL37), which promote dendritic cell activation, with the consequent release of IL-12 and IL-23. These cytokines activate Th1, Th17, and Th22 cells, which produce IFNγ, TNF, IL-17, IL-22, contributing to the pro-inflammatory cytokines' milieu, to which keratinocytes respond (24). These cytokines also play a major role in PsA articular manifestations and, from a genetic standpoint, several cytokines' genes associated with PsO are also associated with PsA (25). However, gene expression patterns in skin and synovium are distinct, showing a stronger IL-17 gene signature in skin and more equivalent TNF and IFNγ gene signatures in both skin and synovium (26). This might explain why treating skin and joints with the same cytokine target may show different grades of efficacy. In the last 20 years, therapeutics that directly target these cytokines (e.g., TNF or IL-17 inhibitors) completely revolutionized PsO treatment, allowing the complete clearing of psoriasis in a number of patients; however, the latter drugs account for PsA disease control only in ≤50% of patients (17). Some patients, for example, might experience diminished response to anti-TNF drugs over time or develop a paradoxical exacerbation of PsO (27); similar cases of paradoxical psoriasis have been reported for secukinumab (anti–IL-17 monoclonal antibody) and ustekinumab (IL-12/23 inhibitor) (28, 29). In recent years, new therapeutics have been developed that offer additional options and a more tailored approach for patients with PsO, some of which are selective cytokines inhibitors, such as guselkumab, risankizumab, and tildrakizumab. These new biological drugs selectively target IL-23, acting upstream in the inflammatory cascade and eventually reducing the production of IL-17 (30). Janus kinase (JAK) inhibitors are new oral small molecules targeting the JAK/signal transducers and activators of transcription (STAT) pathway. Several cytokines, transmit their signals via this pathway, with multiple effects on different cells (31), playing a role in different PsA manifestations, including EAMs and comorbidities (Figure 2). The JAK family consists of JAK1, JAK2, JAK3, and tyrosine kinase 2 (TYK2); inhibition of each of these molecules can interrupt specific STAT-dependent signaling pathways. JAK-dependent cytokines directly and indirectly mediate the inflammatory response in PsO, (IFNγ, IL-12, IL-23 and TNF, IL-17 respectively), thus, a JAK-inhibition acts broader and more upstream in the inflammatory cascade compared with a selective cytokine-inhibition (32). Upadacitinib, a selective JAK1 inhibitor approved for PsA, has shown good results in treating PsO in PsA clinical trials, with 75% improvement in the Psoriasis Area Severity Index achieved (PASI75) even in 52.3% of patients for whom biologic treatment had failed (33). Tofacitinib, a pan-JAK inhibitor (inhibits JAK1/JAK2/JAK3 and to a lesser extent TyK2) has been approved for PsA, and baricitinib, a JAK1/2 selective inhibitor, is being studied as possible treatment for PsO (34). Finally, a selective inhibitor of TYK2 is also under investigation for PsO and PsA^1^, ^2^.

### Inflammatory Bowel Diseases

Crohn's disease (CD) and UC, both IBDs, may occur in patients with PsO and PsA (35). Several studies reported the prevalence of IBD in patients with PsA, ranging from 0 to 20% (3). In a recent meta-analysis, the pooled estimate was 3.3%, although there was major asymmetry on the funnel plot, suggestive of bias (3). An Italian joint consensus by expert rheumatologists, gastroenterologists, and dermatologists has recently defined a core set of red flags for a multidisciplinary referral. Symptoms defined as major criteria for referral to gastroenterologists were bleeding, chronic abdominal pain, perianal fistula or abscess, chronic diarrhea, and nocturnal symptoms. Additionally, authors defined a set of minor criteria, including oral aphtosis, anemia, family history of IBD, weight loss, and fever, underlining the need for at least three of the latter for specialist referral. However, it should be considered that patients with PsO and PsA have a higher risk of developing IBD and that some patients with PsO may have subclinical IBD. To date there is a lack of studies examining the effect of IBD on longitudinal patient-reported outcomes and clinimetrics. The 2019 European Congress of Rheumatology (EULAR 2019) recommendations for PsA treatment (35) therefore advocated a tailored approach, focusing on both musculoskeletal and non-musculoskeletal involvements if present. From a pathogenic standpoint, PsA and IBD seem to share multiple mechanisms.

The interest of the scientific community in a pathogenic link between gut microbiome dysbiosis and PsA (and SpA in general) development has grown in the last few years. Disruption of the normal gut microbiota homeostasis led to systemic inflammation, with a central role of IL-23 in this process, in both SpA and IBD (36). IL-23 behavior, in the context of IBD, is extremely complex because it acts on multiple cells of the innate and adaptive immune systems. Preclinical data in murine models of colitis highlighted the importance of this cytokine in IBD (37); several anti–IL-23p19–specific antibodies, including risankizumab, brazikumab, mirikizumab, and guselkumab, have been or are currently under evaluation in clinical trials in CD and, in some cases, UC (38). Several cytokines sustain and amplify the chronic inflammatory response in IBD, although with different patterns among CD and UC, and many of them are shared by PsA and IBD pathogenesis, with a largely similar therapeutic approach (39). TNF is a crucial cytokine in this context and is considered the main driver of intestinal tissue inflammation; in the last two decades, different TNF blockers have been developed, most of which have been successfully used in IBD and in enteropathic SpA. Still, some patients do not respond to anti-TNF agents or response is lost after one year, mainly because of drug immunogenicity (40). Moreover, anti-TNF inhibitor etanercept is ineffective for IBD (41), and patients with PsA treated with the etanercept had a significant increase in the risk of developing CD [adjusted HR, 2.0 (95% CI, 0.8–2.2)] or UC [2.0 (1.5–2.8)] (42).

CD is mostly a Th1-driven disease compared with UC. This leads to increased levels of IL-12, which acts in synergy with IL-23 and TNF in sustaining the inflammatory response with multiple downstream pathways, including regulation of pro-inflammatory Th17 cells (43). IL-12 and IL-23 share the p40 subunit, which is the target of ustekinumab, a monoclonal antibody approved for CD and also efficient in PsO and peripheral PsA (38). The discovery of the IL-23/Th17 pathways in IBD boosted intensive research aimed at the development of new therapeutics against these targets; unfortunately, both preclinical data and clinical trials showed a paradoxical worsening of the intestinal disease with anti–IL-17 blockage (44). The Janus of the IL-23 and IL-17 neutralization has been recently explained: anti–IL-23 antibodies reduce Th17, improving inflammation, whereas the blockage of IL-17 affects tissue homeostasis repair, impairing intestinal wall integrity and thus exacerbating the disease (45). These negative results have been largely disappointing, especially in the light of a missing option for patients with enteropathic SpA, whose treatment is often more challenging and requires a tighter, tailored, and multidisciplinary approach (46). Similar to PsA, most of the cytokines involved in IBD signal trough the JAK/STAT pathway and multiple clinical trials have recently been initiated to investigate the efficacy of different JAK inhibitors in CD and UC. Indeed, JAK inhibition in these clinical conditions may block multiple cytokines at the same time. Tofacitinib was the first small molecule to be approved for UC, but it was not effective in CD (47, 48). Filgotinib and upadacitinib (the latter of which is already approved for PsA)^3^ are two selective JAK1 inhibitors currently under investigation in phase III clinical trials for CD and UC^4^, ^5^, ^6^, ^7^. These new promising therapeutics might have the advantage of being effective in multiple overlapping clinical conditions, as in the case of enteropathic SpA.

### Uveitis

Ophthalmic manifestations are estimated to occur in 10% of patients with PsO and 31% of patients with PsA (3, 49). Uveitis is the most frequent inflammatory eye involvement. In literature, the prevalence of uveitis is reported to affect between 2 and 25% of patients with PsA (3) and, in a recent meta-analysis of 21 studies, the pooled estimate of uveitis was 3.2% (3). The wide range of prevalence reported in PsA may be explained by the variable sets of classification criteria used for patient selection and the different time of follow-up. Nevertheless, the analysis reported high heterogeneity in analyzed studies and a high risk of bias. Anterior uveitis (AU) is the most frequent clinical phenotype of uveitis in PsA. The reported prevalence of AU ranges from 2 to 25% of cases, and it is more frequently observed in patients with axial PsA or who are HLA-B27–positive (50). The involvement of the anterior chamber prompted Bridgewood et al. (51) to speculate that connective tissue of uveal structures might be conceptually similar to a musculoskeletal enthesis. Indeed, elastin and type IV collagen compose the structure of tendons of the ciliary muscle and IL-23R–positive resident cells have also been recently detected in the ciliary body of mice (51, 52). AU is a potentially vision-impairing condition if not treated. Patients often complain of ocular pain, photophobia, tearing, marked eye redness and, in more severe cases, vision blurring due to abundant inflammatory precipitate in the anterior chamber (53, 54). Although the most common type of uveitis is recurrent AU, it does not appear to follow a “unilateral alternating” pattern of ankylosing spondylitis and both eyes can be affected simultaneously. In an Italian study comparing the frequency of uveitis in PsA with other SpA, PsA uveitis had a more insidious onset, was more frequently bilateral (38 vs. 7%) and posterior (44 vs. 17%) and lasted longer (31 vs. 6%) (55). In a large Italian cross-sectional study involving 278 patients with uveitis (n.418 eyes) who were referred to a rheumatologist in two tertiary care centers, Lopalco et al. (56) reported that AU (63.6%) with a chronic course (71.4%) and bilateral involvement (57.1%) was the most frequent phenotype. Nevertheless, the authors also showed that chronic posterior uveitis (42.8%) with bilateral involvement (66.7%) was likely to occur.

Acute AU has a strong genetic component, and most of the identified susceptibility genes belong to various immunologic pathways that are also common in SpA, including PsA. Besides HLA-B27, other genes involving TNF, IL-17, and IL-23 pathways have been identified for uveitis (57), emphasizing the major role of inflammation in this disease. Studies from animal models revealed a role for both innate and adaptive immunity, in line with SpA pathogenesis (58). Moreover, as with synovial fluid from inflamed joints, several pro-inflammatory cytokines can be found in the aqueous humor in case of uveitis, including TNF, IFNγ, IL-17, IL-22, IL-23 (59). Thus, it is not surprising that biological therapies played a role in the treatment of this disease in the last few years; with their immunomodulatory properties and a more selective immunosuppression, these drugs reduce the need for topical corticosteroids, lowering the risk of elevated intraocular pressure and vision loss (60). Anti-TNF drugs, such as infliximab and adalimumab, can successfully control uveitis flares, whereas etanercept can promote a paradoxical uveitis. The reason for this paradoxical effect is unknown, but it may be linked to a drug-induced cytokine imbalance (61). Other TNF inhibitors, such as golimumab and certolizumab pegol, showed promising results (62, 63), but to date, only adalimumab is indicated for uveitis treatment. Genetic studies and mice models revealed a possible involvement of the IL-23/IL-17 axis as well (64), thus modulation of this pathway may represent a future therapeutic option for this condition. Ustekinumab showed efficacy in some case reports, (65, 66) and a phase II clinical trial has been recently completed^8^. The JAK/STAT pathway is also being studied in uveitis because of its role in cytokines signaling. Topical tofacitinib showed symptom improvement in an experimental model of autoimmune uveitis (67), and phase II clinical trials are currently ongoing to test tofacitinib^9^ and filgotinib in this disease^10^.

## Psoriatic Arthritis Comorbidities

### Cardiovascular Disease, Metabolic Syndrome, and Diabetes

Among the different comorbid conditions that could be present in PsA, cardiometabolic disease is the most prevalent, with an important effect on disease burden and outcomes. A recent work analyzed the incidence of new comorbidities per 100 person-years in PsA patients and controls. Compared with controls, patients with PsA had a higher incidence rate of autoimmune disease, cardiovascular disease, fatigue, eczema, obesity/overweight, depression, anxiety, smoking, cancer, diabetes, alcohol use, osteoporosis, uveitis, and liver disease (68). Furthermore, observational studies showed an increased risk (43%) of cardiovascular diseases in patients with PsA, with higher morbidity risks for myocardial infarction, cerebrovascular diseases, and heart failure compared with the general population (69). Of note, the rates of coronary artery disease hospitalizations were significantly higher in patients with PsA than in controls (primary diagnosis, 0.8 vs. 0.5%; non-primary diagnosis, 3.2 vs. 2.2%; *P* < 0.001 for both) (68). Other studies confirmed the increased incidence of cardiovascular diseases in patients with PsA [incidence rate, 9.4 (95% CI, 6.5–13.5)], and even reports coming from administrative data showed higher risk of cardiovascular disorders (incidence rate, 6.5 vs. 5.8 compared with controls) and a higher risk of specific cardiovascular disorders (hypertension, hyperlipidemia, coronary artery disease, cerebrovascular disease, peripheral vascular disease) (70). All these factors were associated with increased all-cause mortality and, of note, higher cardiovascular mortality rate seemed to be related to the polyarticular pattern, high disease activity, and severity (69). Among cardiovascular risk factors, a recent meta-analysis showed that hypertension is the most prevalent comorbidity in patients with PsA (present in ~39% of patients), followed by hyperlipidemia, diabetes, and obesity (71, 72). Regarding obesity, studies have also suggested that weight gain may be a consequence of the systemic inflammatory state or that obesity may lead to more weight on the joints, altered mechanics, and repetitive micro-trauma, which could represent a trigger for entheseal and synovial inflammation (73). Moreover, obesity is a negative predictor for response to treatment; in particular, response to anti-TNF could be impaired, with higher risk to not achieve the minimal disease activity in obese patients with PsA (74–76). Finally, among the different comorbidities present in patients with PsA, insulin resistance and diabetes mellitus (DM) appeared to be of peculiar interest. DM appears to be more prevalent in patients with PsA compared with general population, with an estimated prevalence of ranging from 6.1 to 20.2%. Moreover, DM seems to be more frequent in patients with PsA vs. patients with only PsO. Clinical factors associated to the development of DM were recently explored: in a large cohort study, tender joint count and erythrocyte sedimentation rate were deemed the predictor factors associated with the development of DM. These results are in keeping with the concept that disease activity is an important driver, suggesting how elevated inflammatory burden may lead to a higher risk of developing DM (6). Indeed, a robust body of evidence indicates a strong bond between the metabolic and the immune system, and alterations in this complex network, may lead to chronic diseases, including diabetes and obesity (77). For example, IL-6, a cytokine renowned for its pro-inflammatory role, is associated with central obesity, hypertension, and insulin resistance. Moreover, it promotes C-reactive protein (CRP) production (78). Additionally, there is a chronic and low-grade state of inflammation called *metaflammation*, described in obesity and type 2 DM, involving the adipose tissue and other tissues (79). As previously discussed, in patients with PsA, the presence of inflammation is associated with an increase in traditional cardiovascular risk factors, such as a higher body mass index, hypertension, high sugar levels, insulin resistance, and dyslipidemia (80). Intensive research in this field brought to light multiple results supporting the hypothesis that the association between PsA and metabolic syndrome might be due to shared inflammatory pathways (81).

#### Role of Cytokines

Increasing evidence has established that PsO, PsA, and atherosclerosis involve the same T-cell–mediated inflammatory pathways, specifically T-helper 1 and T-helper 17 cascades (82), with release of several pro-inflammatory cytokines (e.g., INFγ and TNF), which are involved in the initiation and progression of atherosclerotic plaques in the systemic vasculature. Chronic inflammation in both PsA and atherosclerosis promotes increased production of adipokines and pro-inflammatory cytokines (e.g., TNF) with consequent insulin resistance and endothelial dysfunction (83). Indeed, it has been demonstrated that anti-TNF therapy improves insulin resistance (84). Presence of inflammation also influences lipoprotein levels, and patients with PsA have been shown to have several lipid alterations, including oxidized lipoproteins, which are markers of atherosclerosis. Anti-TNF therapies can increase total cholesterol, high-density lipoprotein (HDL), low-density lipoprotein (LDL), triglycerides, and Apo B levels in patients with PsA and rheumatoid arthritis (RA) without changing the atherogenic index (84). According to some authors, these data suggest normalization with suppression of inflammation; to others, these results might be interpreted with caution despite the reduced cardiovascular risk reported in cohorts of patients treated with anti-TNF (85). Patients with PsA receiving disease-modifying anti-rheumatic drugs (DMARDs) showed a lower cardiovascular risk when compared with those not receiving these medications, and anti-TNF treatment seems to be associated with a reduced progression of subclinical atherosclerosis in these patients (86, 87). TNF is not the only cytokine involved in cardiovascular comorbidity: data from animal models and human studies highlighted the pro-atherogenic role of IL-17 on vascular inflammation, acting in synergy with TNF and IL-6 (88). IL-17 is involved in endothelial dysfunction, hypertension, plaque progression and destabilization, stroke, and myocardial infarction. However, it also seems to have anti-atherogenic effects, and low serum levels of IL-17 have been associated with a higher risk of cardiovascular relapses in patients with coronary artery disease (89). TNF and IL-17 can also inhibit the autophosphorylation of the insulin receptor, thus inducing insulin resistance and suppressing the expression of GLUT4 (90). Another link between PsA and DM can be found in adipokines, a group of cytokines secreted by adipose tissue. Adiponectin is an adipokine with anti-inflammatory, insulin-sensitizing, and anti-atherogenic properties, but whose secretion is decreased by pro-inflammatory cytokines (such as TNF, IL-1β, and IL-6). Some studies have shown that in inflammatory diseases such as PsA, adiponectin levels are decreased, with a potential effect on the metabolic status of patients. Of note, it has been demonstrated that biologic drugs, such as anti-TNF, may increase adiponectin levels, leading to a possible improvement of metabolic aspects in PsA (91, 92). IL-17 also links inflammation with insulin resistance and adipocytes dysfunction; for instance, there is a reciprocal regulation between the pro-inflammatory adipokine leptin and IL-17. In mice models with systemic inflammation and insulin resistance, there is accumulation of IL-6/IL-17 co-expressing T cells in the adipose tissue; these cells enhance leptin production, which eventually acts in synergy with IL-6 and IL-17 to promote Th17 differentiation. This complex network drives local and systemic insulin resistance, and, in fact, IL-17 neutralization improves glucose uptake (93). Moreover, clinical studies in patients with inflammatory arthritis and type II DM demonstrated that anti–IL-17 (and anti–IL-12/23) therapies do not seem to have an influence on body weight and do not increase the risk of DM manifestations (88).

#### Signal Transduction Pathways

Phosphodiesterase-4 (PDE4) is a phosphodiesterase that hydrolyzes cyclic adenosine monophosphate (cAMP) to adenosine monophosphate (AMP). This molecule can be found in keratinocytes and immune cells and is involved in several cytokines' pathways implicated in inflammation. A small molecule targeting PDE4 (apremilast) has been used for the last few years to treat PsO and PsA. Besides inflammatory diseases, derangement of the PDE4-cAMP signaling is relevant in the development of metabolic disorders. In fact, patients with PsA treated with this anti-PDE4 molecule seem to have a better lipid and glucose profile (94). Most of the pro-inflammatory cytokines involved in PsA pathogenesis signal through the JAK/STAT pathway, and a growing body of evidence points to involvement of this pathway in DM and obesity. It has been shown that through activation of the JAK/STAT pathway insulin signal can be decreased, and data have demonstrated that oxidative stress and inflammation work together to induce insulin resistance with JAK performing a central role (95). The β-pancreatic cells respond to insulin, growth factors and cytokines that are JAK/STAT dependent, and this pathway could be involved in both type I and type II DM. In DM mice models (non-obese diabetic [NOD] mice) treatment with a JAK1/JAK2 inhibitor can reverse the disease (96). However, the role of JAK/STAT proteins in metabolism is highly dependent on the context and the cell type. Impairment of JAK/STAT signaling can lead to various metabolic alterations or protection from obesity and insulin resistance. Knock-out mice for JAK3 or TYK2 have been shown to be prone to obesity and insulin-resistance (97, 98). In contrast with these data from animal studies, an *in vitro* study showed that tofacitinib induced “browning” in human adipocytes through IFN suppression (99), in which the increase in the number of brown adipocytes in the adipose tissue has been shown to prevent obesity and improve type II DM. This study opened the path of new area of research, involving JAK inhibitors as possible therapeutic options for obesity (99). Concerning the cardiovascular risk, the effect of JAK inhibitors showed contrasting results. JAK/STAT signaling pathway seems to be deeply involved in atherosclerosis, regulating scavenger receptors involved in LDL uptake (100), and is involved in NOX-dependent oxidative stress in human aortic smooth muscle cells (101). In randomized controlled trials and real-world studies in patients affected by RA, the use of JAK inhibitors tofacitinib, baricitinib, and upadacitinib increased levels of total cholesterol, HDL, and LDL in the first four weeks of therapy and then plateaued, with lack of changes in the atherogenic index (102, 103). *In vitro* studies have shown that this was associated with release of cholesterol from macrophages by reverse cholesterol transport, and, in line with these observations, tofacitinib was shown to ameliorate atherosclerosis in mice models (104). Moreover, in patients with RA treated with upadacitinib, a significantly higher efflux of cholesterol from macrophages was observed, and this was associated with increased HDLs and reduction of CRP (105). Some real-world studies reported increased major cardiovascular events (MACE) and deep venous thrombotic (DVT) events in patients treated with tofacitinib (especially at higher dose) and baricitinib (106) whereas other studies did not support these findings (107, 108). Currently, it is not possible to establish if this increased risk is related to specific direct and indirect cytokine blockade, to chemical structure and/or pharmacologic and toxicologic properties of specific JAK inhibitors, to the presence of concomitant diseases, or other factors (e.g., genetic mutations). The pathophysiological process that eventually leads to blood clot formation, involves the recruitment of a broad variety of immune cells, chemokines, and cytokines, with an inflammatory response that impairs endothelial function and activates the coagulation cascade (109). Inhibition of the JAK/STAT pathway modulates the inflammatory response and, thus, should reduce the prothrombotic risk. According to some authors, JAK-inhibition specificity matters; when a pro-inflammatory and anti-thrombotic pathway is blocked, other pathways can still transmit pro-thrombotic signals, thus, these complications might not be considered a class drug effect (110). The genetic background of patients could also play a big role, especially in terms of age-dependent JAK mutations (110). A good case in point is the mutation V617F in the JAK2 gene, which enhances its function, resulting in an increased risk of thrombotic events. This mutation is frequent among patients affected by myeloproliferative neoplasms, and ruxolitinib (a JAK1/JAK2 inhibitor) is a recommended second-line treatment for the prevention of thrombosis in these patients (111). Another aspect to take into account, is the patients' clinical response to therapy; to the best of our knowledge, there are contrasting data in the literature regarding a correlation between incomplete disease control and increased MACE/DVT events in patients with inflammatory arthritis treated with tofacitinib: a recently published cohort study from Sweden demonstrated a strong association between disease activity and the risk of venous thrombotic events in patients with RA (112); in contrast, a *post hoc* analysis of a phase III trial of tofacitinib in patients with RA, did not find any association between disease activity and the MACE risk, but it revealed a trend toward an association between elevated erythrocyte sedimentation rate following tofacitinib treatment with an increased risk of future MACE, and this was explained as possibly a form of failure to respond to treatment (113). Thus, the persistence of inflammation, together with other predisposing factors, may contribute to the development of these events in certain individuals.

### Osteoporosis

In PsA, bone involvement is quite complex because it involves not only bone loss but also new bone formation. According to some studies, prevalence of osteoporosis (OP) in patients with PsA is similar to the general population (114). However, other studies have shown that OP is found very frequently in PsA, ranging from between 1.4 and 68.8% of patients (9–12), but its likelihood depends on the site of measurement, the arthritic subset, the sex of the patient, and other factors (e.g., menopausal state) (115). Moreover, the use of glucocorticoids are also a contributing factor, although to a lesser extent than RA, requiring a different type of OP management compared with the general population (114). OP is associated with an increased risk of fractures and, according to a study form Pedreira et al. prevalence of fractures seems higher in patients with PsA versus PsO and controls, despite no differences in bone mineral density in the three groups (116). This finding highlights the concept that not only bone density but also bone quality matters when dealing with increased fracture risk (115). The role of the immune system in bone quality and bone metabolism is well established and led to the development of an intriguing field of research called osteoimmunology (117). Bone undergoes continuous remodeling thanks to a delicate but dynamic balance between osteoclasts and osteoblasts. The immune cells and the neuro-endocrine system regulate these cells, answering to physiological/mechanical stress, and dysregulation of this network leads to various bone diseases, including bone damage in inflammatory arthritis and OP (118). Several studies described the role of inflammation in OP pathogenesis as involving both the innate and the adaptive immune systems. Moreover, the presence of a low-grade, chronic, systemic, inflammatory state, associated with aging, has been linked to age-related diseases, including OP (119). Cytokines involved in PsA pathogenesis have an effect on bone cell activity with possible inhibitory or stimulatory stimuli on osteoclasts and osteoblasts. TNF, IL-6, and IL-1, for example, exert stimulatory activity toward osteoclasts and inhibitory activity toward osteoblasts (119). Given the activity of these inflammatory cytokines on bone cells, the high prevalence of OP in a systemic chronic inflammatory disease such as PsA is not surprising. IL-12 and IL-23, involved in PsA, are also critical to inflammation-induced bone resorption. Specifically, IL-23 upregulates the receptor activator of nuclear factor kappa-B (RANK) on preosteoclasts and induces Th17 cells to produce IL-17 (120). IL-17, one of PsA signature cytokines, promotes bone resorption via RANK ligand upregulation (121). Indeed, Th17 cells have been found to be highly increased in blood and tissues of patients with OP (72). PsA and OP often share another risk factor that is tightly connected to inflammation: vitamin D deficiency. A possibility of crosstalk between vitamin D and IL-33, a cytokine involved both in PsA and OP, has recently been suggested (122). Vitamin D and IL-33 under some conditions act in synergy and under other conditions modulate each other. For instance, they both have a protective effect on bone resorption, whereas in inflammatory conditions, vitamin D deficiency and IL-33 upregulation boost each other (123). The relationship between inflammation and bone loss suggests that, in light of their immunomodulatory properties, biologic drugs can reduce OP and fracture risk (124). Most of research on this topic focuses on the role of anti-TNF drugs, and several of these studies demonstrated a role of these therapeutics in bone loss reduction in patients with inflammatory arthritis (125, 126), although other studies did not completely confirm these data (127). The effect of TNF-blockade on fracture risk specifically still needs to be fully elucidated. Indeed, a recent article by Manara and Sinigaglia concluded that there is little evidence of a clinically relevant effect of TNF-inhibitors on this, especially considering the conflicting results published in literature (125). Similarly, the effect of IL-17 and IL-23 blockade on bone loss is still not completely clear. Animal studies support a bone protective effect of anti-IL-23 and anti-IL-17 antibodies, preventing bone loss (128, 129). However, clinical studies, especially in the context of inflammatory arthritis, are missing. Most of these cytokines control bone homeostasis via JAK/STAT proteins (130) in a tightly regulated way in physiologic conditions. In cases of inflammation, pathologic activation of this pathway can eventually lead to OP, bone erosions, and other bone disorders. Therefore, inhibition of this pathway, which exerts a positive effect on prevention of bone erosions (131), may have a similar effect on bone density (132, 133). In fact, recently published papers indicated that JAK/STAT inhibition induces osteoanabolic effects in mice models and *in vitro* studies (134, 135).

### Mood Disorders

Current evidence suggests that patients with PsA have a significantly worse quality of life compared with patients experiencing other rheumatic diseases (136). This may be due to the additive effect of PsO on chronic pain, limitations in physical functioning and work abilities, extreme fatigue, and emotional and social impairment. Such a detrimental effect on quality of life would also entail depression and anxiety in patients with PsA (7, 8). Indeed, according to a recent meta-analysis, the prevalence of depression in patients with PsA ranges from 9 to 22%, and the prevalence of anxiety between 15 and 30%, which is higher than in the general population (137). Anxiety and depression in PsA are more likely to affect patients who are female, unemployed, and with high disease activity (8). The 2019 EULAR recommendations (35) emphasized that comorbidities should be taken into account in the management of PsA, highlighting that anxiety and depression are among the most common ones in such patients. This is particularly important in planning a treat-to-target strategy because the presence of comorbid depression and anxiety has recently been shown to reduce the likelihood of achieving disease remission, according to the American College of Rheumatology/European League Against Rheumatism Boolean and Disease Activity Index for PsA Norwegian-DMARD prospective cohort (138). There is rising evidence that an inflammatory process might be also behind these diseases. Specifically, IL-6 might have a negative effect on mood, and it has been shown to be a predictor of higher severity and chronicity of depression (139). Moreover, elevated levels of CRP and TNF have been found in patients with depression and have been associated with higher symptoms severity (139). There is evidence that neuroinflammation also plays a role in depression, with increased pro-inflammatory cytokines in the brain and activation of microglia cells (140). Anti-inflammatory treatment has been shown to improve symptoms of depression according to different studies (141). However, contrasting evidence has been shown regarding the effect of treatment with conventional synthetic and biological DMARDs on psychological disorders. One study demonstrated a reduction in prevalence of depression and anxiety with etanercept treatment (142), but these findings are at high risk of bias because of insufficient data on sampling, response rates, and statistical analysis. According to a recent meta-analysis (137), the overall effect of PsA treatment on depression and anxiety remained unclear. Lastly, patients treated with a PDE4 inhibitor for PsA and PsO showed increased frequency of depression or suicidal behavior in clinical studies (143).

### Fibromyalgia

Fibromyalgia (FM) is a chronic syndrome characterized by pain and tenderness, sleep disturbance, fatigue, cognitive dysfunction, and background emotional distress. It sometimes presents as a comorbidity with another disease or condition (144). FM is the clinical expression of stress-related neurobiological responses that lead to increased reactivity in several sensory neural systems, particularly those in the musculoskeletal system. In most patients with comorbid FM, such responses may be related to the burden of having a chronic illness. This includes the symptoms of the disease; the effect on general health; the need for tests, drugs, and treatments; disability; loss of quality of life; and changes in social and work roles (144). In the general population, the prevalence of FM varies, according to classification criteria, between 2 and 8%. These rates increase significantly when FM manifests as comorbidity, particularly in rheumatic diseases, with FM symptoms often merging with those of the underlying condition. Data from retrospective cohorts show that the prevalence of classified FM in patients with PsA according to 2016 American College of Rheumatology criteria is higher, ranging from 17.8 to 64% (7, 145–147). FM can either interfere with the patient's perception of disease activity or alter physicians' clinimetrics. Indeed, patients with FM frequently experience widespread pain that can be mistaken for arthralgia or enthesitis. Comorbid FM is known to amplify the perception of pain and fatigue in patients with PsA and therefore, negatively affects self-reported assessment of disease activity. Furthermore, coexistent FM can make the therapeutic strategy for PsA challenging hampering the global clinical effectiveness of therapies and misleading doctors in their choices. In general, patients with PsA with FM are more likely to be female with polyarticular phenotype and high disease activity score, whichever index is adopted (145–147). Elsawy et al. and Ulutatar et al. (146, 147) report that patients with PsA with comorbid FM show a significant increase in disease activity scores that incorporate measurements of pain and tenderness compared with patients without FM. These include standard instruments such as the Disease Activity in PsA Index (DAPSA), Tender Joint Count, Leeds Enthesitis Index, and Disease Activity Score on 28 Joints (148). Furthermore, patients with PsA with FM had significantly poorer sleep quality, greater fatigue, and lower quality of life (146, 147). Of note, the FM PsA group had also a significantly higher body mass index, which is another factor potentially influencing clinical response to treatment (17). Patients with PsA with FM receiving biologic treatments may also have a significantly lower response rate than those without FM at all time points in terms of both DAPSA remission and minimal disease activity (145). Interestingly, the time to discontinuation of the first and second biologic drugs was also far shorter in patients with PsA with versus without FM, with the FM diagnosis doubling the risk of discontinuing treatment (145).

Fibromyalgia may also affect patient-reported outcomes, such as the Health Assessment Questionnaire Disability Index (HAQ-DI) and the 12-item PsA Impact of Disease Questionnaire (PsAID-12). Several authors agree that patients with FM PsA have higher (worse) HAQ-DI scores than those without PsA (145, 147). The presence of a coexisting FM might also influence PsAID-12 interpretation (149). Fibromyalgia has long been considered a non-inflammatory rheumatic disease, but multiple recent studies have brought to light the possible role of inflammation in FM pathogenesis (150). Immunologic alterations are added to genetic, hormonal, environmental, and neural factors and can contribute to the development of an inflammatory state (151). Interestingly, a very recent genetic study investigated the gene expression profile in patients with FM, finding that most of modulated genes belonged to the IL-17 pathway and to the type I IFN signatures, suggesting an autoimmune component for this disease (152). These data corroborate the findings of increased serum levels of pro-inflammatory cytokines, such as IL-1, IL-6 and IL-17, found in patients with FM (153). All of these cytokines are known to be involved in the neuroinflammation contributing to pain also found in inflammatory arthritis, including PsA. For example, IL-1 induces sensory neural sensitization to pain, probably trough tyrosine kinases (154); IL-6 is released by hepatocytes during pain stimuli and by neurons and glial cells and has been associated to hyperalgesia, depression, fatigue and sympathetic nervous system activation (153); and IL-17 modulates pain by increasing nociceptor excitability (155). This has empowered the idea of targeting pro-inflammatory cytokines as a potential therapy for FM; however there are no data currently available in the literature on the possible use of biological drugs in this condition. A key mechanism behind FM and chronic pain is believed to be central sensitization (CS). CS is a phenomenon of synaptic plasticity and increased neuronal responsiveness involving the central nervous system (CNS) (156). CS is typically characterized by activation of glia cells and astrocytes with release of cytokines and chemokines, and accumulating evidence suggests that this neuroinflammation process promotes chronic widespread pain in the body via CS (156). Activation of glia cells and astrocytes in neuroinflammation occurs via activation of the intracellular JAK/STAT signaling pathway (157); this pathway has a pleiotropic effect in the context of CNS, being involved in the regulation of multiple neural functions (158). Several pre-clinical data described the role of this pathway in amplification of pro-inflammatory cytokines implicated in neuropathic pain (154), and the new generation of JAK inhibitors demonstrated rapid pain relief (159). Given the role of this pathway and their dependent and independent cytokines in CS, it is possible to speculate a positive effect of JAK inhibitors on central chronic pain and, thus, FM symptoms, although further studies are needed to demonstrate this hypothesis.

## Conclusions

Psoriatic arthritis is a multifaceted disease encompassing different domains and is associated with several comorbidities that must be considered in the clinical management of patients. The systemic inflammation involved in the musculoskeletal domain plays a major role in EAMs and comorbidities. Current available immunomodulatory treatments can have a multilayered, *yin and yang* effect, which underscores the need for therapies with a more comprehensive disease target.

## Author Contributions

LN: conceived the work, reviewed the literature, and wrote the manuscript. EL, FI, VV, and FP: reviewed the literature and wrote the manuscript. FM and GC: critically contribute to the idea development, reviewed, and wrote the manuscript. All authors contributed to the article and approved the submitted version.

## Funding

AbbVie participated in the design of the project, as well as in interpretation of the data, review, and approval of the publication.

## Conflict of Interest

FM, LN, and GC, are AbbVie employees and may own AbbVie stocks/options. FI has received consultancy fees and/or speaker honoraria from Actelion, Pfizer, AbbVie, Janssen, Celgene, Novartis, MSD, BMS, UCB, Sobi, Roche, Sanofi outside this work. The remaining authors declare that the research was conducted in the absence of any commercial or financial relationships that could be construed as a potential conflict of interest.

## Publisher's Note

All claims expressed in this article are solely those of the authors and do not necessarily represent those of their affiliated organizations, or those of the publisher, the editors and the reviewers. Any product that may be evaluated in this article, or claim that may be made by its manufacturer, is not guaranteed or endorsed by the publisher.

## Footnotes

^1^Available online at: https://clinicaltrials.gov/ct2/show/NCT04772079?term=deucravacitinib&draw=2&rank=3.

^2^Available online at: https://clinicaltrials.gov/ct2/show/NCT03881059.

^3^Available online at: https://www.ema.europa.eu/en/documents/smop/chmp-post-authorisation-summary-positive-opinion-rinvoq-ii-04ii-05_en.pdf.

^4^Available online at: https://www.clinicaltrials.gov/ct2/show/NCT02914600?term=filgotinib&cond=crohn+disease&draw=2&rank=1.

^5^Available online at: https://www.clinicaltrials.gov/ct2/show/results/NCT02914522?term=filgotinib&cond=Ulcerative+Colitis&draw=2&rank=2.

^6^Available online at: https://www.clinicaltrials.gov/ct2/show/NCT03653026?term=upadacitinib&cond=Ulcerative+Colitis&draw=2&rank=1.

^7^Available online at: https://www.clinicaltrials.gov/ct2/show/NCT03345836?term=upadacitinib&cond=Crohn+Disease&draw=2&rank=1.

^8^Available online at: https://clinicaltrials.gov/ct2/show/results/NCT02911116.

^9^Available online at: https://clinicaltrials.gov/ct2/show/results/NCT03580343?term=tofacitinib&cond=Uveitis&draw=2&rank=1.

^10^Available online at: https://clinicaltrials.gov/ct2/show/NCT03207815?term=filgotinib&cond=Uveitis&draw=2&rank=1.

## Abbreviations

AMP: adenosine monophosphate
AU: anterior uveitis
cAMP: cyclic adenosine monophosphate
CD: Crohn disease
CNS: central nervous system
CRP: C-reactive protein
CS: central sensitization
DAPSA: Disease Activity in PsA Index
DM: diabetes mellitus
DMARD: disease-modifying anti-rheumatic drug
DVT: deep venous thrombotic
EAM: extra-articular manifestation
FM: fibromyalgia
HAQ-DI: Health Assessment Questionnaire Disability Index
HDL: high-density lipoprotein
HR: hazard ratio
IBD: inflammatory bowel disease
IFN: interferon
IL: interleukin
JAK: Janus kinase
LDL: low-density lipoprotein
MACE: major cardiovascular event
OP: osteoporosis
PDE4: phosphodiesterase-4
PsA: psoriatic arthritis
PsAID-12: 12-item PsA Impact of Disease Questionnaire
PsD: psoriatic disease
PsO: psoriasis
RA: rheumatoid arthritis
RANK: receptor activator of nuclear factor kappa-B
SpA: spondyloarthritis
STAT: signal transducers and activators of transcription
TNF: tumor necrosis factor
TYK2: tyrosine kinase 2
UC: ulcerative colitis.
